# Soil Contamination from PCB-Containing Buildings

**DOI:** 10.1289/ehp.9646

**Published:** 2006-11-06

**Authors:** Robert F. Herrick, Daniel J. Lefkowitz, George A. Weymouth

**Affiliations:** 1 Department of Environmental Health, Harvard School of Public Health, Boston, Massachusetts, USA; 2 pcbinschools.org, Yorktown, New York, USA; 3 International Union of Bricklayers and Allied Craft Workers, Local 3 (Retired), Boston, Massachusetts, USA

**Keywords:** caulk, environmental exposure, leachability, PCB, polychlorinated biphenyl, public buildings, schools, soil

## Abstract

**Background:**

Polychlorinated biphenyls (PCBs) in construction materials, such as caulking used around windows and expansion joints, may constitute a source of PCB contamination in the building interiors and in surrounding soil. Several studies of soil contamination have been conducted around buildings where the caulking has been removed by grinding or scraping. The PCBs in soil may have been generated in the process of removing the caulking, but natural weathering and deterioration of the caulking may have also been a source.

**Objectives:**

The objectives of this study were to measure PCB levels in soil surrounding buildings where PCB-containing caulk was still in place, and to evaluate the mobility of the PCBs from caulking using the Toxicity Characteristic Leaching Procedure (U.S. Environmental Protection Agency Method 1311).

**Discussion:**

We found soil PCB contamination ranging from 3.3 to 34 mg/kg around buildings with undisturbed caulking that contained 10,000–36,200 mg/kg PCBs. The results of the Toxicity Characteristic Leaching Procedure (leachate concentrations of 76–288 mg PCB/L) suggest that PCBs in caulking can be mobilized, apparently as complexes with dissolved organic matter that also leach off the caulking material.

**Conclusions and Recommendations:**

Although these new findings are based on a small sample size, they demonstrate the need for a national survey of PCBs in building materials and in soil surrounding these buildings. Because the buildings constructed during the time the PCB caulking was in use (1960s and 1970s) include schools, hospitals, and apartment buildings, the potential for exposure of children is a particular concern. It is necessary to reconsider the practice of disposing of old PCB caulking removed during building renovations in conventional landfills, given the apparent mobility of PCBs from the caulking material. Disposal of some caulking material in nonhazardous landfills might lead to high PCB levels in landfill leachate.

In June 2004, we reported the results of a study in which we found elevated levels of polychlorinated biphenyls (PCBs) in building caulking materials used around windows and in expansion joints in masonry buildings (Herrick et al. 2004). Our investigation of 24 buildings in the Greater Boston (Massachusetts) area revealed that one-third (8 of 24) contained caulking materials with PCB content > 50 ppm (mg/kg) by weight. The U.S. Environmental Protection Agency (EPA) considers materials exceeding 50 ppm PCB that were not specifically authorized for use by the U.S. EPA to be “unauthorized-use” nonliquid PCB products that require removal and decontamination (U.S. EPA 1998). PCB bulk product waste is defined as

waste derived from manufactured products containing PCBs in a non-liquid state, at any concentration where the concentration at the time of designation for disposal was ≥ 50 ppm PCBs.… PCB bulk product waste includes, but is not limited to … [n]on-liquid bulk wastes or debris from the demolition of buildings and other man-made structures manufactured, coated, or serviced with PCBs. (U.S. EPA 1998)

Results similar to those found in Boston have been reported by investigators examining buildings in Germany, Finland, and Sweden (Balfanz et al. 1993; Burkhardt et al. 1990; Coghlan et al. 2002; Corner et al. 2002; Fromme et al. 1996; Pyy and Lyly 1998). In Switzerland, a national survey focused on concrete (masonry) buildings found that almost half of all such buildings erected between 1955 and 1975 (1,348 buildings sampled) contained joint sealants (caulking) with PCB concentrations of 20–550,000 mg/kg (Zennegg et al. 2004).

Priha et al. (2005) sampled soil around 11 buildings from which PCB-containing caulking had been removed and reported total PCB concentrations in soil of 0.11–26.9 mg/kg. The highest PCB concentrations were in areas closest to the buildings, and they declined as the distance increased. The average PCB concentration in samples taken within 2 m of the buildings was 6.83 mg/kg; at 3–10 m from the walls, it was 0.52 mg/kg. The highest soil concentrations were found on the southern side of the buildings (average concentrations: south, 16.6 mg/kg; west, 2.00 mg/kg; east, 2.39 mg/kg; north, 3.96 mg/kg). Priha et al. (2005) concluded that

The area south of these buildings is more contaminated than those in other directions, and, therefore, the weathering of sealants is probably an important mechanism in the spread of PCBs to the surroundings.

However the old caulking had been abated in these buildings. Grinding of the old caulking material to remove it from the building masonry joints could also have contributed to the finding of soil contamination.

At a New York State elementary school constructed in 1969, PCB-containing caulking (60,000 mg/kg) was removed during window replacement in 2003 (Whitaker 2005). Wipe samples were taken inside and outside the school. Indoor sampling locations included classroom floors, walls, and windows as well as inside the ventilation system. Outdoor sampling locations consisted mainly of windows, but also included the surrounding soil to determine the contamination into the surrounding environment. Surface concentrations on the outside of the building measured via wipe samples ranged from 0.92 μg/100 cm^2^ (alcove above the boiler room) to 34 μg/100 cm^2^ (outdoor window sill). The U.S. EPA (1998) considers a surface to be contaminated if concentrations exceed 10 μg/100 cm^2^. Whitaker (2005) reported PCB levels in indoor wipe samples ranging from 0.22 μg/100 cm^2^ (classroom) to 2.3 μg/100 cm^2^ (plenum of exhaust system). Soil contamination of 0.96–40.0 mg/kg (eight samples) was also found. The caulking material in other parts of the building was not replaced, although it was visibly deteriorated (an example from the present study is shown in Figure 1). As in the case of the Finnish buildings studied by Priha et al. (2005), the soil contamination could have been caused by weathering of the caulking, but the generation of PCB-containing particles during scraping and grinding to remove the old caulking material could not be ruled out as a source.

For the present study our objectives were to measure PCB levels in soil surrounding buildings where PCB-containing caulk was still in place, and to evaluate the mobility of the PCBs from caulking using the Toxicity Characteristic Leaching Procedure [U.S. EPA Method 1311 (U.S. EPA 1992)].

## Methods

We identified three buildings (designated A, B, and C) where PCB-containing caulk appeared to be present. In the opinion of an experienced bricklayer (G.W.) who examined these buildings, the PCB-containing caulking had not been disturbed or removed from the walls we selected for sampling. These three buildings were typical of masonry buildings constructed in the 1960s and 1970s. One was a university family-housing unit, and the other two were schools. We sampled the caulking, and at each building we also sampled surface soil at a distance of approximately 30 cm from the building foundation. PCB content of both the caulking and soil samples was determined in accordance with U.S. EPA Method 8082 (U.S. EPA 2000).

In order to assess the mobility of PCBs from samples of caulking material, we used the Toxicity Characteristic Leaching Procedure [TCLP; U.S. EPA Method 1311 (U.S. EPA 1992)]. Of the three buildings where we had collected paired caulking and soil samples, we had only the recommended amount of caulking material (100 g) from building A to conduct the procedure, so we selected 2 other caulking samples from the original set of 24 that we tested in 2004 (Herrick et al. 2004). This test was designed to simulate leachate generation from a material if it were co-disposed with municipal solid waste in a nonhazardous waste landfill (U.S. EPA 1998). The extraction liquid simulates municipal solid waste leachate. Although these are not the conditions to which intact caulking would be subjected during natural weathering in a building, this test does determine the mobility of analytes in liquid, solid, and multiphasic waste; it is used to determine whether PCB bulk product waste can be disposed of in nonhazardous waste landfills. We postulated that the finding of PCBs in the caulking leachate would suggest a possible pathway between the caulking material and soil. Each of these three caulking samples had PCB content > 5,000 mg/kg in the bulk material.

## Results

The analysis of the bulk caulking material from the three buildings yielded the following results: building A, 36,200 mg/kg; building B, 10,000 mg/kg; building C, 14,800 mg/kg. Soil analysis for PCB in the soil surrounding these buildings found 34 mg/kg at building A; 3.3 mg/kg at building B; and 3.4 mg/kg at building C.

Caulking samples from the three buildings subjected to the TCLP contained 36,200 mg/kg (building 1, which was the same as building A), 5,010 mg/kg (building 2), and 5,970 (building 3). Analysis of the extract from the three samples analyzed by the TCLP found PCBs at 76 mg/L (building 1), 137 mg/L (building 2 ), and 288 mg/L (building 3). These levels exceed by a factor of at least 7,600 the 10-μg/L limit for the result of leachate tests that allows PCB bulk product waste to be disposed of in nonhazardous waste landfills (U.S. EPA 1998).

## Discussion

In 2004, we found that 8 of 24 buildings sampled in the Greater Boston Area contained caulking material with > 50 ppm PCB, with the highest level of 36,200 ppm (Herrick et al. 2004). The findings from studies in Finland (Priha et al. 2005) and the investigation at the PCB-containing school in New York (Whitaker 2005) strongly suggest that this caulking material can be a source of soil contamination around the outside perimeter of these buildings. In these cases, however, the caulking had been removed from the buildings before testing for soil contamination. Because the process of removing the caulking includes scraping, grinding, and other steps that may aerosolize the PCB-containing material, the source of the soil contamination could not be established. Natural weathering and deterioration of the caulking over the almost 30 years it was in the building walls may have contributed, but soil contamination from the removal process could not be ruled out.

In the present study, we selected walls of buildings where the caulking had apparently never been disturbed. We found PCB soil contamination around these buildings, and the results of the TCLP demonstrate that PCBs appear to be readily mobilized from the caulking. The TCLP results should be interpreted with caution because the procedure is designed to simulate conditions in municipal solid waste landfills and not natural weathering. Because the PCB concentrations in the extracts from the TCLP far exceed the aqueous solubility of PCBs (generally around 0.1–10 μg/L, depending on the congener), we believe that the PCBs apparently exist as complexes with dissolved organic matter that also leached off the caulking material.

**Figure 1:**
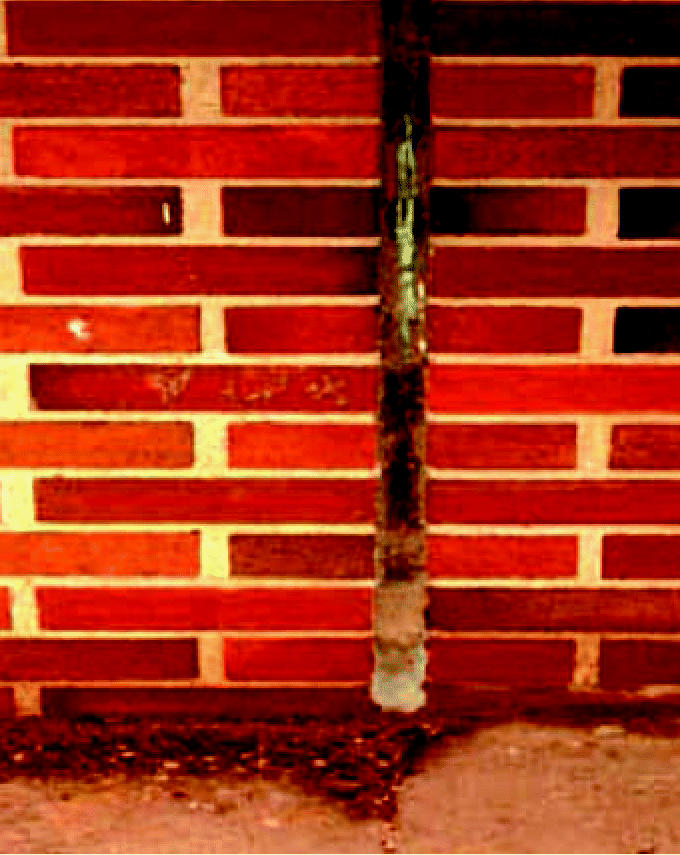
Deteriorated PCB caulking in a building expansion joint.

Although the concentration of PCBs in the bulk caulking samples appeared to be a reasonable predictor of the amount of PCBs found in the soil around the buildings containing the caulking, the amount of PCBs released by the TCLP extraction procedure was not related to the PCB content of the bulk material. This may be a result of the small number of samples we examined. Given that the caulking material is at least 30 years old, it may be degraded to the point that the PCBs, which were plasticizers in the original polysulfide polymer formulations, can be mobilized into solution. In some cases, the caulking has clearly lost its elasticity (Figure 1) and the extent of degradation in any caulking material sample may be a better predictor of the amount of PCBs that can be mobilized than the bulk PCBs content of the caulk.

## Conclusions

Our findings suggest that the most likely cause of soil contamination found around these PCB-containing buildings is natural weathering. PCBs appear to be mobilized from the caulking as part of a complex with dissolved organic material. The practice of disposing of old PCB caulking removed during building renovations in conventional landfills should be reconsidered, given the apparent mobility of PCBs from the caulking material. Disposal of this caulking material in nonhazardous waste landfills could lead to high PCB levels in landfill leachate. In 2004 we recommended a random probability-based survey of schools, hospitals, and other masonry buildings constructed or renovated during the time PCB-containing caulking was in use, to assess the extent to which this material is still in place (Herrick et al. 2004). Although our study is small, these new findings suggest that this survey should include measurement of PCBs in soil surrounding buildings where PCB caulking is present and an assessment of the risk that this material may pose, especially to children in schools and other buildings where soil contamination is found.
